# *DUSP1* promoter methylation in peripheral blood leukocyte is associated with triple-negative breast cancer risk

**DOI:** 10.1038/srep43011

**Published:** 2017-02-21

**Authors:** Jing Li, Yanbo Chen, Hongyuan Yu, Jingshen Tian, Fengshun Yuan, Jialong Fan, Yupeng Liu, Lin Zhu, Fan Wang, Yashuang Zhao, Da Pang

**Affiliations:** 1 Department of Epidemiology, School of Public Health, Harbin Medical University, Harbin, Heilongjiang Province, P. R. China; 2 Department of Breast Surgery, The Affiliated Tumor Hospital of Harbin Medical University, Harbin, Heilongjiang Province, P. R. China

## Abstract

DNA methylation is one of the most common epigenetic alterations, providing important information regarding cancer risk and prognosis. A case-control study (423 breast cancer cases, 509 controls) and a case-only study (326 cases) were conducted to evaluate the association of *DUSP1* promoter methylation with breast cancer risk and clinicopathological characteristics. No significant association between *DUSP1* methylation in peripheral blood leukocyte (PBL) DNA and breast cancer risk was observed. *DUSP1* methylation was significantly associated with ER/PR-negative status; in particular, triple-negative breast cancer patients showed the highest frequency of *DUSP1* methylation in both tumour DNA and PBL DNA. Soybean intake was significantly correlated with methylated *DUSP1* only in ER-negative (OR 2.978; 95% CI 1.245–7.124) and PR negative (OR 2.735; 95% CI 1.315–5.692) patients. Irregular menstruation was significantly associated with methylated *DUSP1* only in ER-positive (OR 3.564; 95% CI 1.691–7.511) and PR-positive (OR 3.902, 95% CI 1.656–9.194) patients. Thus, *DUSP1* methylation is a cancer-associated hypermethylation event that is closely linked with triple-negative status. Further investigations are warranted to confirm the association of environmental factors, including fruit and soybean intake, irregular menstruation, and ER/PR status, with *DUSP1* methylation in breast tumour DNA.

Breast cancer is the most common cancer among women worldwide. The World Health Organization reported that there were 1.67 million new breast cancer cases and 0.52 million deaths attributed to breast cancer worldwide in 2012, while in the same year in China, newly diagnosed cases and deaths totalled 187,000 and 48,000, respectively^1^. According to latest estimates, 246,660 new female breast cancer cases and 40, 450 cancer deaths are projected to occur in the United States in 2016^2^.

Among many signalling pathways, the mitogen-activated protein kinase (MAPK) cascades are central to cell proliferation and apoptosis. The first MAPK phosphatase to be identified was mitogen-activated protein kinase phosphatase-1 (MKP-1), which is encoded by the dual-specificity phosphatase 1 (*DUSP1*) gene and mediates the dephosphorylation of MAPKs^3^. MKP-1 is an endogenous inhibitor of the mitogen-activated protein kinase (MAPK) pathway through inhibiting the activation of ERK^4^,^5^. Although the mechanisms of MAPK signalling pathways in breast cancer development, progression, and tamoxifen resistance have been well-documented^6^,^7^,^8^,^9^, very little is known about the role of MKP-1 in breast carcinogenesis. Accumulating evidence has shown reduced MKP-1 mRNA or protein expression in several types of cancers including prostate^10^, epithelial^11^, renal^12^ and urothelial^13^ cancers. Chen *et al*.^14^ suggested there was a significant reduction in *DUSP1* mRNA expression in five breast cancer cell lines compared with a normal control.

Carcinogenesis is a multi-stage process driven by the accumulation of genetic and epigenetic abnormalities^15^. DNA methylation is a critical mechanism of epigenetic modification involved in gene expression programming. Abnormal DNA methylation occurs primarily in CpG islands within gene promoters, resulting in transcriptional inactivation and gene silencing, and contributes to the tumorigenesis of several cancers^16^,^17^,^18^. It has been proposed that the methylation status of some CpG sites could be passed on from previous generations as an inherited marker^19^. Several studies have been conducted on the changes in DNA methylation in blood leukocyte DNA, and suggested a link of blood leukocyte DNA methylation with cancer susceptibility^20^,^21^,^22^,^23^. Ji-Yeob *et al*. found that leukocyte DNA hypomethylation is independently associated with the development of breast cancer^24^. Thus, peripheral blood leukocyte (PBL) DNA might be a potential surrogate biomarker for cancer risk assessment. In addition, epigenetic variation can arise as a consequence of environmental, dietary, and aging factors^25^,^26^,^27^,^28^. Different tissues may exhibit different responses to environmental factors, and methylation status in leukocytes may not fully reflect the changes in the target tissue^29^. Methylation–environment interactions may provide further explanations for the complexity of cancer development.

Genome-wide differing methylated regions have been detected by comparing breast tumour tissue DNA and adjacent normal tissue DNA using next-generation sequencing techniques^30^. Several promising methylated biomarkers have been identified from circulating cell-free DNA between breast cancer cases and controls^31^,^32^,^33^,^34^. Specific methylation patterns were proposed to correlate with distinct clinicopathological characteristics^35^,^36^ and assist in identifying individuals who will respond to therapy and survive longer. Hence, with suitable assays and validation in large populations, such associations can be exploited in non-invasive diagnosis and personalized treatment decisions.

Given the lack of research on *DUSP1* methylation in breast cancer in epidemiological studies, we first investigated the association between *DUSP1* methylation in PBL DNA, interactions with environmental factors, and breast cancer risk. We also explored the correlation between clinicopathological characteristics and *DUSP1* methylation in both tumour DNA and PBL DNA, as well as the effect of environmental factors on *DUSP1* methylation in tumour tissue DNA.

## Results

### Association between *DUSP1* methylation in PBL DNA and breast cancer risk

PBL DNA was extracted from 423 patients and 509 controls. Supplemental Table 1 shows the distribution of demographic characteristics in cases and controls. No significant difference was found for age (*P* = 0.276) and BMI (*P* = 0.154). However, there were significant differences for the distribution of marital status (*P* = 0.023), educational level (*P* = 0.002), occupation (*P* = 0.001), and family history of cancer (*P* = 0.000) between cases and controls. Hence, these four variables were adjusted in the subsequent multivariate analyses.

*DUSP1* methylation was detected in 5.2% (22/423) breast cancer cases and 4.9% (25/509) controls in PBL DNA (Table 1). After adjusting for marital status, educational level, occupation, and family history of cancer, no significant difference in *DUSP1* methylation was observed between cases and controls. Therefore, we cannot conclude any association between *DUSP1* methylation in PBL DNA and breast cancer risk (OR 0.79, 95% CI 0.414–1.504, *P* = 0.472).

### Association of *DUSP1* methylation in PBL DNA and environmental factors on breast cancer risk

Supplemental Table 3 shows the univariate and multivariate logistic regression analyses for all associations between environmental factors and breast cancer risk. Several environmental factors, including the consumption of refined grains, vegetables, fruit, seafood, milk, smoked food, etc., were found to be associated with the development of breast cancer following adjustment for educational level, occupation, marital status, and family history of cancer. We analysed the interactions of *DUSP1* methylation with all of the above significant environmental factors. However, no significant interaction was observed (as shown in Table 1). Therefore, we concluded that there was not enough evidence for *DUSP1* methylation in PBL DNA as a biomarker for breast cancer risk assessment.

### Differences in *DUSP1* methylation frequency between tumour DNA and PBL DNA in breast cancer patients

Genomic DNA from 326 breast tumour tissue samples was detected for *DUSP1* methylation: the positive frequency of *DUSP1* methylation was 59.2% (193/326). We successfully detected *DUSP1* methylation in both PBL DNA and tumour DNA from 155 breast cancer patients. As shown in Table 2, a total of 83 tumour DNA were methylated in 155 tumour samples with a methylation frequency of 53.55%; in contrast, only five (3.23%) PBL DNA was methylated among the same patients. The *P*-value (0.000) from the McNemar Test indicates that there was a significant difference for the *DUSP1* methylation frequency between the DNA samples from these two tissue types.

### Correlation between clinicopathological characteristics and *DUSP1* methylation in breast tumour DNA and PBL DNA

As show in Table 3, similar significant associations of ER and PR status and molecular subtypes with *DUSP1* methylation in tumour DNA and PBL DNA were observed. Aberrant methylation of *DUSP1* occurred more frequently in tumour DNA (OR = 2.278, 95% CI 1.389–3.735, *P* = 0.001) and PBL DNA (OR = 2.534, 95% CI 1.062–6.044, *P* = 0.036) with oestrogen receptor (ER)-negativity, as well as for progesterone receptor (PR)-negativity in tumour DNA (OR = 2.016, 95% CI 1.275–3.186, *P* < 0.01) and PBL DNA (OR = 3.034, 95% CI 1.264–7.282, *P* = 0.013). In particular, in a molecular subtype analysis, compared with luminal A breast cancer (ER and/or PR + , HER2-), patients with HER2-enriched (ER and PR-, HER2 + ) and basal-like (ER-, PR-, HER2-) subtypes showed significantly higher *DUSP1* methylation frequencies with ORs of 2.661 (95% CI 1.345–5.267, *P* = 0.005) and 5.636 (95% CI 2.205–14.406, *P* = 0.000), respectively, in tumour DNA; patients with the basal-like (ER-, PR-, HER2-) subtype showed significantly higher *DUSP1* methylation frequency with an OR of 5.238 (95% CI 1.108–24.763, *P* = 0.000) in PBL DNA, which indicated that *DUSP1* methylation is linked with ER/PR-negative status and is a significant characteristic of triple-negative breast cancer.

No significant association of *DUSP1* methylation in tumour DNA with TNM stage, tumour invasion, lymph node involvement, metastasis status, histological type, or *TP53* mutation status was observed.

### Effect of exposure to environmental factors on *DUSP1* methylation in tumour tissue DNA

Supplemental Table 2 shows the distribution of demographic characteristics between patients with methylated and unmethylated tumour DNA. No statistically significant difference was observed for any demographic characteristic (all *P* > 0.05).

We analysed the associations between environmental factors and *DUSP1* methylation in tumour DNA in a multivariate analysis. Among all the environmental factors, there was no significant effect on *DUSP1* methylation status from menopause, breast massage, breast hyperplasia, breast disease, smoking, alcohol etc. (Supplemental Table 4). However, the consumption of fruit and soybean, and irregular menstruation significantly correlated with the *DUSP1* methylation status of tumour DNA in both univariate and multivariate analyses (Table 4). Individuals with a lower fruit intake (≤ 1000 g/week) had a higher proportion (67.9%) of methylated *DUSP1*. Individuals with a higher soybean intake (>1 times/week) and irregular menstruation had higher proportions of methylated *DUSP1* with OR of 1.955 (*P* = 0.006) and 2.000 (*P* = 0.020), respectively.

Since soybean intake and irregular menstruation are two hormone-related factors, the associations between soybean intake, menstrual cycle and *DUSP1* methylation were further examined by dividing the subjects according to ER and PR status. As show in Table 5, we found significant data for soybean intake and irregular menstruation amongst the subgroups. Soybean intake (>1 times/week) was significantly correlated with increased *DUSP1* methylation only in patients with ER-negative (OR 2.978, 95% CI 1.245–7.124) and PR-negative (OR 2.735, 95% CI 1.315–5.692) breast cancer. Meanwhile, irregular menstruation was significantly linked with increased *DUSP1* methylation only in patients with ER-positive (OR 3.564, 95% CI 1.691–7.511) and PR-positive (OR 3.902, 95% CI 1.656–9.194) breast cancer.

## Discussion

Several studies have suggested that individual variation in the epigenome in blood is associated with aging and environmental factors encountered throughout life^37^,^38^, with consequent risk of breast^39^, ovarian^40^, bladder^20^, and small-cell lung cancer^41^. The potential utility of specific methylation biomarkers in PBL DNA as novel markers of cancer susceptibility has been proposed. In this study, we first explored the value of *DUSP1* methylation in PBL DNA for the risk assessment of breast cancer, but we failed to find any association between *DUSP1* methylation in PBL DNA with breast cancer risk, or for the interactive effects of *DUSP1* methylation and environmental factors. However, we did find significant correlations of triple-negative status with *DUSP1* methylation in both tumour DNA and PBL DNA, and significant associations among soybean intake, irregular menstruation, ER/PR status, and *DUSP1* methylation in tumour DNA.

Increasing numbers of studies have identified tissue-specific differential methylation^42^, which will provide important novel insights into normal and pathogenic mechanisms, as well as help in identifying markers of carcinogenesis and future epigenetic therapies. In this study, we analysed differences in *DUSP1* promoter methylation between PBL DNA and tumour DNA. A significantly higher *DUSP1* methylation frequency was observed in tumour DNA than in PBL DNA. In the research of Chen *et al*., they identified a normal breast cell line (M10) that was completely unmethylated while several breast cancer cell lines (MCF7, MDA-MB-231, SKBR3, and BT474) exhibited 100% methylation; unmethylation was dominant (86.2%) in benign breast tumours whereas methylation was dominant (57.2%) in invasive breast tumours^14^. Together, we concluded that *DUSP1* methylation is a cancer-associated hypermethylation event. The biological significance of *DUSP1* hypermethylation in breast cancer should be addressed in future *in vitro* studies. Given the very low (3.23–5.2%) methylation frequency in PBL DNA, *DUSP1* methylation has potential as a biomarker in non-invasive breast cancer diagnosis if we can detect *DUSP1* methylation from circulating cell-free DNA in plasma that is released by breast tumour cells.

Some genes that exhibit special methylation status in tumours are correlated with ER/PR status. ER-positive tumours were found to be more frequently methylated on *RASSF1A* than ER-negative tumours^43^. PR had a positive correlation with *DUSP1* expression in 30 human breast cancer cell lines by binding to two progesterone response elements downstream of the *DUSP1* transcriptional start site to upregulate *DUSP1* promoter activity^44^. A critical novel finding of this study was the linkage of ER/PR-negativity with methylated *DUSP1* in both breast tumour DNA and PBL DNA, which might account for the lower MKP-1 expression.

It is well known that negative results for oestrogen receptor (ER-), progesterone receptor (PR-), and HER2 (HER2-) expression in breast cancer cells signifies that the cancer is triple-negative cancer. These negative results indicate that the growth of the cancer is not supported by the oestrogen and progesterone hormones, or by the presence of too many HER2 receptors. Therefore, triple-negative breast cancer does not respond to hormonal therapy (such as tamoxifen or aromatase inhibitors) or therapies that target HER2 receptors. In contrast, ER-positive and PR-positive tumours are associated with improved response to hormonal therapy and with a longer disease-free interval and improved survival^45^,^46^. Doctors and researchers have an intense interest in developing their understanding of triple-negative breast cancer pathogenesis and finding new medications that can treat this breast cancer type. Holm *et al*. determined the methylation status of 807 breast cancer-related genes according to molecular subtype and found that basal-like, luminal A and luminal B tumours have different methylation profiles^47^. In this study, we found triple-negative breast tumour showed the highest frequency of *DUSP1* methylation. Hence, *DUSP1* methylation might be considered as a distinctive subtype-specific marker of triple-negative patients.

Most established risk factors for female breast cancer are thought to influence the susceptibility to cancer through hormone-related pathways^48^. Epidemiological experimental evidence implicated that increased concentrations of endogenous oestrogen level or exogenous oestrogen intake may induce aberrant DNA methylation^49^. In this study, we found high intake of fruit was correlated with decreased *DUSP1* methylation, while high intake of soybean and irregular menstruation were correlated with increased *DUSP1* methylation.

Soybean is a unique food because it contains large amounts of isoflavones^50^,^51^. Isoflavones have a chemical structure that is very similar to the hormone oestrogen^52^. To the best of knowledge, breast cancer is a heterogeneous disease and biological differences in subtypes depend on the expression of receptors, including ER, PR, and HER2. Because of the ability of isoflavones to bind oestrogen receptors, the varied associations between soybean intake and breast cancer risk by the hormone receptor status of tumours have been suggested in eight published epidemiological studies^53^,^54^,^55^,^56^,^57^,^58^,^59^,^60^. However, few studies have researched the modified effect of soybean intake on DNA methylation, which may have pivotal functions in relation to tumour suppression, apoptosis, etc. in breast cancer. Harlid *et al*. used an Illumina Human Methylation450 BeadChip to evaluate epigenome-wide DNA methylation in vaginal cells from soy formula-fed and cow formula-fed girls; the results indicated that girls fed soy formula had altered DNA methylation in their vaginal cell DNA^61^. Only one study has been published that provides evidence on the potential effects of two naturally occurring isoflavones, genistein and daidzein, on the methylation of *BRCA1* and *BRCA2* tumour suppressor genes in breast cancer cell lines (MCF-7, MDA-MB 231, and MCF10a)^62^. Our study is the first to explore the putative effects of soybean consumption on *DUSP1* promotor methylation in breast cancer. We found that soybean intake was significantly associated with methylated *DUSP1* in tumour DNA in ER/PR-negative patients. Messina and colleagues reviewed substantial epidemiological data from observational studies and cell culture data, and noted that the current limited knowledge regarding the effect of soybean on breast cancer-related issues suggested that clinicians should be careful of what they prescribe for patients^63^,^64^. Although information regarding soybean consumption was provided as retrospective data for the patients’ dietary habit prior to cancer diagnosis, given the significant correlation between soybean intake and *DUSP1* methylation we observed, prolonged or excessive consumption of soybean in ER/PR-negative patients is not recommended.

In addition, progesterone balances oestrogen and in doing so minimises the negative effects of oestrogen–progesterone imbalances^65^. When these hormones become unbalanced it is usually because of oestrogen dominance, which means too much oestrogen compared with the levels of progesterone. Irregular menstruation flow is an oestrogen-dominant symptom. In the subgroup analyses in our study, irregular menstruation was significantly correlated with increased *DUSP1* methylation in ER/PR-positive patients, which suggested that irregular menstruation may correlate with *DUSP1* methylation through the indirect effect of oestrogen. Further *in vitro* studies are needed to validate this inference.

There are some limitations with the interpretation of the present results. First, some studies have suggested cell-specific variation in DNA methylation^66^,^67^; in our case-control study design, we only focused on the differential methylation of *DUSP1* in leukocyte DNA between cases and controls, and we did not further explore the cell type composition difference of PBL between the cases and controls. Second, our conclusion for the association between ER/PR status and *DUSP1* methylation in breast tumour DNA was generated based on a population study without an experimental validation *in vitro* study. Third, the small number of subjects in the stratified analysis limited the statistical power to evidence the conclusion; further studies with larger sample sizes are encouraged to verify the relationship between environmental factors, ER/PR status, and *DUSP1* methylation.

## Conclusions

The results of this study indicated that *DUSP1* methylation in PBL DNA and its interaction with environmental factors was not associated with breast cancer risk. This is the first study to report the modified effects of soybean consumption on DNA methylation in tumour by ER/PR status, and we provide preliminary evidence on potential epigenetic changes through *DUSP1* methylation in triple-negative patients. Further validation of the association of environmental factors, including fruit and soybean intake, and irregular menstruation, with *DUSP1* methylation by hormone receptor status in breast cancer should be undertaken.

## Materials and Methods

### Study subjects

We carried out this study after obtaining informed written consent from study subjects and approval from the Human Research and Ethics Committee of Harbin Medical University. All experiments including all relevant details were performed in accordance with relevant guidelines and regulations.

A case-control study was designed to assess the role of *DUSP1* methylation and interactions with environmental factors on breast cancer risk. All breast cancer patients were newly diagnosed cases recruited from the Third Affiliated Clinical Hospital of Harbin Medical University from 2010 to 2014. Controls were recruited from patients admitted to the Orthopaedic and Ophthalmology of the Second Affiliated Hospital of Harbin Medical University, and volunteers from the Xiangfang community of Harbin City within the same time period. Any individual with a history of benign breast disease or any other cancer was excluded from the control group. Approximately 5 ml of peripheral venous blood was obtained from all cases either before surgery for the patients and at enrolment for the controls.

A case-only study was designed to explore the difference in *DUSP1* methylation between breast tumour DNA and PBL DNA. Tumour tissues specimen were collected during surgery and rapidly frozen in liquid nitrogen after removal, then returned to the lab and stored at −80 °C immediately. We analysed the correlation between clinicopathological characteristics and *DUSP1* methylation in tumour DNA and PBL DNA, as well as the effect of exposure to environmental factors on *DUSP1* methylation in tumour DNA.

### Data collection

All subjects were interviewed face-to-face by well-trained interviewers using the same questionnaires, which included questions on demographic information (age, marital status, education, occupation, family cancer history, height and weight), behaviours (smoking, drinking, physical activity), dietary status (intake of milk, vegetables, fruits, soy bean etc.) during the 12 months prior to cancer diagnosis, menstruation and reproductive history, and any other disease history. The clinical and pathological information of cancer patients was extracted from medical records, including TNM stage, histological, and pathological results.

### Genomic DNA extraction

PBL DNA was extracted from blood samples using a commercial DNA extraction kit (QIAamp DNA Blood Mini Kit, Hilden, Germany) according to the manufacturer’s protocol and then stored at −80 °C. Less than 25 mg of minced tumour tissue was used for DNA extraction. Tumour tissues were removed from the deep freeze and ground into small pieces immediately by a tissue grinder. DNA was extracted from tumour tissues using a DNA extraction kit (PureLinkTM Genomic DNA Kit, Carlsbad, USA) according to the manufacturer’s protocol and then stored at −80 °C. DNA quantity was measured using the Nanodrop 2000 Spectrophotometer (Thermo Scientific).

### Sodium bisulphite modification

Bisulphite conversion was performed using 2 μg DNA and an EpiTect Bisulfite Kit (Qiagen, Hilden, Germany) according to the manufacturer’s guidelines. DNA yield after bisulphite conversion was in the range of 50–100 ng/μl; DNA was stored at −80 °C.

### Analysis of the methylation status of *DUSP1*

Methylation-sensitive high-resolution melting analysis (MS-HRM) was performed on a LightCycler 480 (Roche Applied Science, Mannheim, Germany) equipped with Gene Scanning software (version 2.0) to detect and analyse the methylation status of *DUSP1*^68^. Universal methylated and unmethylated DNA standards (ZYMO, USA) were used as the positive and negative controls. To create the range of methylated and unmethylated allele dilutions, the above two standards were mixed at 1, 5, 10, and 20% ratios.

Primers were designed for MS-HRM analysis using Primer Premier 5.0 software as follows: forward primer, 5′-TGGTTTGGTAGGGCGGGTGA-3′, and reverse primer, 5′–GTCGCACACACAACCCAAATA-3′. The PCR product (range = chr5:172198165–172198336, 171 bp) was located at CpG island IV located on the border of the promoter and exon 1 of *DUSP1*. There is an Illumina 450 K probe within this region (cg11757894 = chr5:172197877), as shown in Supp. Fig. 1. PCR reactions were performed using LightCycler 480 ResoLight Dye (Roche Applied Science), primers at 200 nmol/L final concentration, 3 nmol/L MgCl_2_ for *DUSP1*, and 5 ng of bisulphite-converted DNA sample in 10 μl final volume.

The PCR amplification protocol consisted of denaturation for 10 min at 95 °C for one cycle, denaturation for 10 s at 95 °C, annealing with a touchdown (65–55 °C, 30 s, in tumour DNA; 70–64 °C, 40 s, in PBL DNA) of each primer annealing temperature and extension for 10 s at 72 °C for 58 cycles. The HRM melting protocol then consisted of 95 °C for 1 min, cool down to 40 °C for 1 min, 70 °C for 5 s and continuous acquisition to 90 °C at 20 acquisitions per 1 °C (LightCycler480, Roche, Mannheim, Germany). We repeated the MS-HRM assay for the DNA samples without a good application curve.

**Figure 1:**
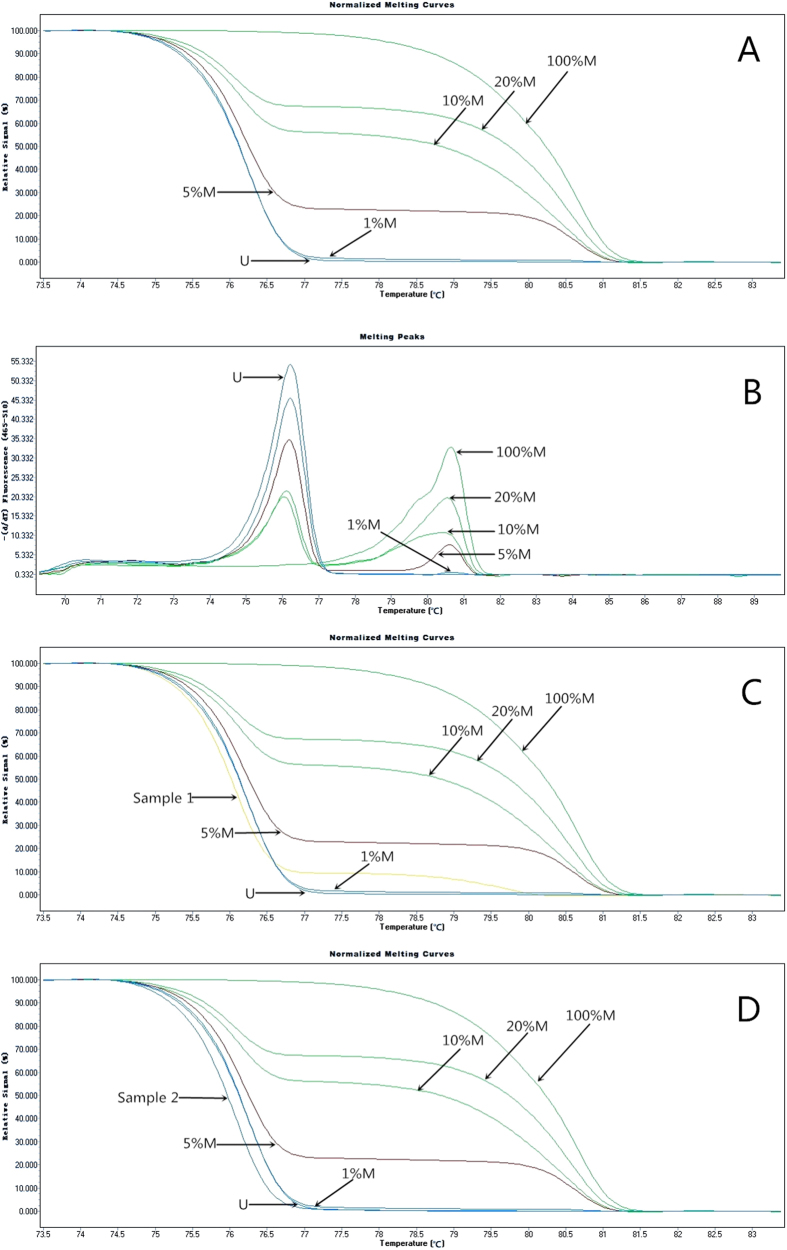
MS-HRM of the *DUSP1* promoter methylation for serials standards and samples. (**A**) Normalized HRM curves. The DNA methylation standards of 0 (universal unmethylated DNA), 1, 5, 20, and 100% methylation (universal methylated DNA) are indicated. (**B**) *T*_m_ plot (negative first derivative of the HRM curves) of serials standards. (**C**) The melting profile of a methylated breast tumour DNA sample (sample 1 with methylation level of 1–5%). (**D**) The melting profile of an unmethylated tumour DNA sample (sample 2).

Then, 0% M (universal unmethylated DNA standards) served as the cut-off value to distinguish methylation and non-methylation of *DUSP1*. We analysed methylation as a qualitative variable, methylated (any methylated status with methylation level higher than 0%M) and unmethylated. We duplicated sample DNA, two blank controls, and gradient methylated DNA standards in each plate. Figure 1(a,b) showed the profile of fluorescence obtained at the melting temperature for serial dilutions of methylated DNA (100%, 20%, 10%, 5%, 1%, and 0%). Figure 1(c,d) showed the melting profiles of a methylated breast tumour DNA and a unmethylated sample.

### Immunohistochemical assay

The presence of oestrogen receptor (ER), progesterone receptor (PR), and HER2 in breast tumour tissue was tested by immunohistochemical (IHC) assay; further verification using fluorescence *in situ* hybridization (FISH) was needed if the results of IHC assays showed HER2-positivity.

### Statistical analysis

Categorical and continuous variables were tested by chi-square test and two-sample t-test, respectively. Univariate and multivariate logistic-regression analyses were used to calculate the crude and adjusted odds ratios (ORs) and 95% confidence intervals (CIs) for the association of environmental factors, *DUSP1* methylation in PBL DNA, and their association with breast cancer risk.

Correlation between clinicopathological characteristics and *DUSP1* methylation status in tumour DNA and PBL DNA was evaluated using odds ratios (ORs) and 95% CIs derived from unconditional logistic regression. The effect of environment factors on *DUSP1* methylation in tumour DNA was calculated using unconditional univariate and multivariate logistic regression.

All statistical analyses were performed using SAS version 9.2, with *P*-values of < 0.05 considered statistically significant.

## Additional Information

**How to cite this article**: Li, J. *et al*. *DUSP1* promoter methylation in peripheral blood leukocyte is associated with triple-negative breast cancer risk. *Sci. Rep.* **7**, 43011; doi: 10.1038/srep43011 (2017).

**Publisher's note:** Springer Nature remains neutral with regard to jurisdictional claims in published maps and institutional affiliations.

## Supplementary Material

Supplemental Tables

## Figures and Tables

**Table 1:** Association of *DUSP1* methylation in peripheral blood leukocyte DNA and environmental factors on breast cancer risk.

Factors	Cases No. (%)	Controls No. (%)	OR_crude_ (95%CI)	*P*-value	OR_adj_ (95%CI)	*P*-value	Interaction	
							OR_i_ (95%CI)	*P*-value
	423	509						
*DUSP1* methylation								
Methylated	22 (5.2)	25 (4.9)	1.000		1.000			
Unmethylated	401 (94.8)	484 (95.1)	0.790 (0.414–1.504)	0.472	1.247 (0.540–2.878)	0.605		
Refined grain (g/day)								
<100	260 (57.4)	290 (42.6)	1.000		1.000		1.000	
≥100	90 (25.7)	215 (97.2)	0.434 (0.308–0.610)	0.000	0.424 (0.275–0.652)	0.000	2.169 (0.463–10.163)	0.326
Vegetable (g/day)								
<500	283 (68.2)	261 (52.0)	1.000		1.000		1.000	
≥500	132 (31.8)	241 (48.0)	0.657 (0.487–0.888)	0.006	0.374 (0.241–0.582)	0.000	0.581 (0.156–2.167)	0.419
Fruit (g/week)								
<1500	216 (52.4)	291 (58.3)	1.000		1.000		1.000	
≥1500	196 (47.6)	208 (41.7)	1.569 (1.164–2.114)	0.003	1.775 (1.172–2.687)	0.007	2.030 (0.491–8.403)	0.328
Garlic (times/week)								
<4	50 (11.8)	288 (56.6)	1.000		1.000		1.000	
≥4	173 (41.7)	221 (43.4)	0.228 (0.157–0.332)	0.000	0.233 (0.143–0.380)	0.000	0.589 (0.056–6.151)	0.659
Seafood (times/month)								
<1	378 (91.3)	421 (83.2)	1.000		1.000		1.000	
≥1	36 (8.7)	85 (16.8)	0.528 (0.325–0.857)	0.010	0.431 (0.223–0.832)	0.012	1.260 (0.185–8.602)	0.814
Milk (times/week)								
<3	310 (74.0)	300 (59.8)	1.000		1.000		1.000	
≥3	109 (26.0)	202 (40.2)	0.487 (0.349–0.679)	0.000	0.402 (0.252–0.641)	0.000	1.439 (0.350–5.910)	0.614
Health care products								
No	244 (58.5)	35 (72.3)	1.000		1.000		1.000	
Yes	173 (41.5)	13 (27.7)	1.685 (1.224–2.320)	0.001	2.356 (1.505–3.689)	0.000	0.455 (0.113–1.837)	0.269
Overnight Food (times/week)								
≤3	239 (57.2)	350 (70.4)	1.000		1.000		1.000	
>3	179 (42.8)	147 (29.6)	1.934 (1.422–2.632)	0.000	1.745 (1.152–2.643)	0.009	1.172 (0.298–4.603)	0.821
Sports								
No	270 (64.4)	270 (53.7)	1.000		1.000		1.000	
Yes	149 (35.6)	233 (46.3)	0.490 (0.362–0.665)	0.000	0.447 (0.294–0.681)	0.000	1.552 (0.415–5.807)	0.514
Menstrual cycle								
regular	342 (82.2)	45 (90.7)	1.000		1.000		1.000	
irregular	74 (17.8)	47 (9.3)	1.891 (1.218–2.936)	0.005	2.510 (1.429–4.410)	0.001	0.332 (0.046–2.420)	0.277
Breast massage								
No	185 (46.7)	36 (74.0)	1.000		1.000		1.000	
Yes	211 (53.3)	127 (26.0)	3.746 (2.720–5.159)	0.000	3.961 (2.594–6.047)	0.000	0.502 (0.136–1.858)	0.302
Mammary gland hyperplasia medication history								
No	332 (79.4)	488 (96.3)	1.000		1.000		1.000	
Yes	86 (20.6)	19 (3.7)	6.013 (3.416–10.582)	0.000	3.692 (1.811–7.526)	0.000	0.296 (0.040–2.200)	0.234
Contraceptive ring								
No	105 (25.1)	157 (30.9)	1.000		1.000		1.000	
Yes	313 (74.9)	351 (69.1)	1.443 (1.040–2.002)	0.028	1.563 (1.001–2.442)	0.050	0.697 (0.181–2.678)	0.599

OR_crude_, odds ratio generated by univariate logistic regression; OR_adj_, odds ratio generated by multivariate logistic regression; OR_i_, odds ratio generated by multivariate logistic regression for the interaction of *DUSP1* methylation and environmental factors; 95%CI, 95% confidence interval.

**Table 2:** Differences in *DUSP1* methylation frequency between tumour DNA and PBL^a^ DNA in breast cancer patients.

PBL DNA^a^	Tumour tissue DNA		Total No. (%)	*P*-value^b^
	Methylated No. (%)	Unmethylated No. (%)		
Methylated No. (%)	3 (1.94)	2 (1.29)	5 (3.23)	0.000
Unmethylated No. (%)	80 (51.61)	70 (45.16)	150 (96.77)	
Total No. (%)	83 (53.55)	72 (46.45)	155 (100)	

^a^PBL, peripheral blood leukocytes. ^b^*P*-value was generated by McNemar Test.

**Table 3:** Correlation between clinicopathological characteristics and *DUSP1* methylation in breast tumour DNA and PBL DNA^a^.

Clinicopathologic characteristics	*DUSP1* methylation (tumour tissue DNA)				*DUSP1* methylation (PBL DNA^a^)			
	Methylated No. (%)	Unmethylated No. (%)	OR_crude_ (95% CI)^b^	*P*-value	Methylated No. (%)	Unmethylated No. (%)	OR_crude_ (95% CI)^b^	*P*-value
TNM Stages				0.886				0.327
I	38 (60.3)	25 (39.7)	1.000		8 (8.0)	92 (92.0)	1.000	
II	113 (59.8)	76 (40.2)	0.978 (0.546–1.751)	0.941	11 (4.7)	221 (95.3)	0.572 (0.223–1.469)	0.246
III & IV	42 (56.8)	32 (43.2)	0.863 (0.436–1.709)	0.674	3 (3.3)	87 (96.7)	0.397 (0.102–1.543)	0.182
Tumour invasion				0.566				0.110
T0–T1	86 (61.0)	55 (39.0)	1.000		13 (7.3)	166 (92.7)	1.000	
T2–T4	107 (57.8)	78 (42.2)	1.140 (0.729–1.782)		9 (3.7)	234 (96.3)	0.491 (0.205–1.176)	
Lymphnodes involved				0.506				
N0	103 (60.9)	66 (39.1)	1.000		11 (5.2)	201 (94.8)	1.000	
N1/N3	90 (57.3)	67 (42.7)	1.162 (0.747–1.808)		11 (5.3)	197 (94.7)	1.020 (0.432–2.408)	
Metastasis status				0.950				0.999
M0	186 (59.2)	128 (40.8)	1.000		22 (5.4)	389 (94.6)		
M1	7 (58.3)	5 (41.7)	1.038 (0.322–3.343)		0 (0.0)	11 (100.0)		
Histological type				0.243				0.871
Invasive	143 (57.4)	106 (42.6)	1.000		17 (5.3)	303 (94.7)	1.000	
Noninvasive	50 (64.9)	27 (35.1)	1.373 (0.807–2.335)		5 (4.9)	97 (95.1)	0.919 (0.330–2.556)	
ER status				**0.001**				**0.036**
Positive	113 (52.8)	101 (47.2)	1.000		12 (3.8)	301 (96.2)	1.000	
Negative	79 (71.8)	31 (28.1)	2.278 (1.389–3.7335)		10 (9.2)	99 (90.8)	2.534 (1.062–6.044)	
PR status				**0.003**				**0.013**
Positive	94 (51.9)	87 (48.1)	1.000		9 (3.2)	271 (96.8)	1.000	
Negative	98 (68.5)	45 (31.5)	2.016 (1.275–3.186)		13 (9.2)	129 (90.8)	3.034 (1.264–7.282)	
HER2 expression				0.503				0.779
Positive	127 (60.5)	83 (39.5)	1.000		15 (5.4)	261 (94.6)	1.000	
Negative	64 (56.6)	49 (43.4)	0.854 (0.537–1.357)		7 (4.8)	139 (95.2)	1.141 (0.455–2.865)	
Molecular subtype^c^				**0.001**				0.108
Luminal A	33 (44.0)	42 (56.0)	1.000		3 (2.7)	110 (97.3)	1.000	
Luminal B	81 (57.0)	61 (43.0)	1.690 (0.961–2.971)	**0.068**	9 (4.4)	197 (95.6)	1.675 (0.444–6.317)	0.446
HER-2 enriched	46 (67.6)	22 (32.4)	2.661 (1.345–5.267)	**0.005**	6 (8.5)	65 (91.5)	3.385 (0.819–13.944)	0.092
Basal-like	31 (81.6)	7 (18.4)	5.636 (2.205–14.406)	**0.000**	4 (12.5)	28 (87.5)	5.238 (1.108–24.763)	**0.037**
P53				0.649				0.151
Positive	49 (61.2)	31 (38.8)	1.000		8 (8.1)	91 (91.9)	1.000	
Negative	143 (58.4)	102 (41.6)	0.887 (0.529–1.487)		14 (4.3)	308 (95.7)	0.517 (0.210–1.271)	

^a^PBL, peripheral blood leukocytes; ^b^OR_crude_, odds ratio generated by univariate logistic regression; 95%CI, 95% confidence interval. ^c^Subtypes were classified by immunohistochemical surrogates as basal-like (ER-, PR-, HER-2−, triple-negative), luminal A (ER and/or PR+, HER-2−), luminal B (ER and/or PR+, HER-2+), or HER-2 enriched (ER and PR−, HER-2+).

**Table 4:** Effect of exposure to environmental factors on *DUSP1* methylation in tumour DNA.

Environmental factors (%)	*DUSP1* methylation		OR_crude_ (95% CI)	*P*-value	OR_adj_ (95% CI)	*P*-value
	Methylated	Un-methylated				
Fruit (g/week)				**0.021**		**0.023**
≤1000	89 (67.9)	42 (32.1)	1.000		1.000	
>1000	100 (54.9)	82 (45.1)	0.576 (0.360–0.920)		0.567 (0.348–0.924)	
Soybean (times/week)				**0.007**		**0.006**
≤1	72 (51.8)	67 (48.2)	1.000		1.000	
>1	119 (66.9)	59 (33.1)	1.877 (1.189–2.962)		1.955 (1.211–3.154)	
Menstrual regularity				**0.014**		**0.020**
regular	134 (55.8)	106 (44.2)	1.000		1.000	
irregular	54 (72.0)	21 (28.0)	2.034 (1.156–3.578)		2.000 (1.113–3.593)	

OR_crude_, odds ratio generated by univariate logistic regression; OR_adj_, odds ratio generated by multivariate logistic regression; 95%CI, 95% confidence interval.

**Table 5:** Association of environmental exposures and *DUSP1* methylation in tumour DNA by ER and PR status.

Environmental factors (%)	ER−			ER+			PR−			PR+		
	Meth^a^	Unmeth^b^	OR_adj_^c^ (95%CI) *P*-value	Meth^a^	Unmeth^b^	OR_adj_^c^ (95%CI) *P*-value	Meth^a^	Unmeth^b^	OR_adj_^c^ (95%CI) *P*-value	Meth^a^	Unmeth^b^	OR_adj_^c^ (95%CI) *P*-value
Soybean (times/week)												
≤1	29	19	1.000	43	47	1.000	36	27	1.000	36	39	1.000
>1	50	11	2.978 (1.245–7.124) 0.017	68	48	1.548 (0.889–2.696) 0.159	62	17	2.735 (1.315–5.692) 0.010	56	42	1.444 (0.789–2.643) 0.282
Menstrual regularity												
Yes	58	21	1.000	75	84	1.000	69	31	1.000	64	74	1.000
No	19	10	0.688 (0.276–1.716) 0.475	35	11	3.564 (1.691–7.511) 0.001	27	13	0.933 (0.425–2.047) 0.863	27	8	3.902 (1.656–9.194) 0.001

^a^Meth, methylated; ^b^Unmeth, unmethylated; ^c^OR_adj_, odds ratio generated by multivariate logistic regression; 95%CI, 95% confidence interval.
