# Supplementary material for: DUSP1 promoter methylation in peripheral blood leukocyte is associated with triple-negative breast cancer risk
Source: Sci Rep. 2017 Feb 21;7:43011. doi: 10.1038/srep43011 (PMC5318948; doi:10.1038/srep43011)
Supplement: Supplemental Tables [file srep43011-s1.pdf]

Title Page

DUSP1 promoter methylation in peripheral blood leukocyte is associated with triple-negative breast cancer

Jing Li[1#], Yanbo Chen[2#], Hongyuan Yu[1], Jingshen Tian[1], Fengshun Yuan[1], Jialong Fan[1], Yupeng Liu[1], Lin Zhu[1], Fan Wang[1*], Yashuang Zhao[1*], Da Pang[2*]

[1]Department of Epidemiology, School of Public Health, Harbin Medical University, Harbin, Heilongjiang Province, P. R. China.

[2]Department of Breast Surgery, The Affiliated Tumor Hospital of Harbin Medical University, Harbin, Heilongjiang Province, P. R. China.

[#]**First author**: Jing Li and Yanbo Chen are co-first authors of this paper.

[*]**Corresponding author**:

Fan Wang, Department of Epidemiology, School of Public Health, Harbin Medical University. 157 Baojian Rd, Nangang District, Harbin Medical University, Harbin, Heilongjiang Province, P. R. China.Tel: 0086-(0)451-86611797. Fax: 0086-(0)451-87502885. E-mail: yifan.701@163.com

Yashuang Zhao, Department of Epidemiology, School of Public Health, Harbin Medical University. 157 Baojian Rd, Nangang District, Harbin Medical University, Harbin, Heilongjiang Province, P. R. China. Tel: 0086-(0)451-86611797. Fax: 0086-(0)451-87502885. E-mail：zhao_yashuang@263.net.

Da Pang, Department of Breast Surgery. The Affiliated Tumor Hospital of Harbin Medical University. 150 Haping Road, Nangang district, Harbin, Heilongjiang Province,

P. R. China. Tel: 0086-(0)451-86298613. Fax:0086-(0)451-86663760. E-mail：

pangda@ems.hrbmu.edu.cn

Supplemental Table 1

Demographic characteristics of breast cancer patients and controls

Characteristics	Cases No. (%)	Controls No. (%)	*P*-value
	423	509	
Age			
<40	43 (10.2)	61 (12.0)	
40-49	145 (34.3)	163 (32.0)	0.276
50-59	149 (35.2)	160 (31.4)	
≥60	86(20.3)	125(24.6)	
Marital status[a]			
single	46 (10.9)	34 (6.7)	**0.023**
married	376 (89.1)	473 (93.3)	
Education level[a]			
Primary and below	99 (23.4)	148 (29.2)	
Junior high school	147 (34.8)	167 (32.9)	**0.002**
Senior middle school	106 (25.1)	145 (28.6)	
College and above	71(16.8)	47 (9.3)	
Occupation[a]			
Mental worker	103 (26.9)	85 (17.4)	
Manual worker	139 (36.3)	226 (46.2)	**0.001**
Mixed	141 (36.8)	178 (36.4)	
Family history of cancer[a]			
Yes	117 (28.1)	71 (15.8)	**0.000**
No	300 (71.9)	379 (84.2)	
BMI[a] (kg/m^2)			
≤18.5	11 (2.6)	21 (4.1)	
18.5-23.0	180 (42.8)	190 (37.5)	0.154
≥23.0	230 (54.6)	296 (58.4)	

[a]Missing values: Marital status 3; BMI 4; Education level 2; Occupation 60; Family history of cancer 65.

Supplemental Table 2

Demographic characteristics of breast cancer patients with tumour tissues

**Characteristics**	***DUSP1* methylation status**		***P*-value**
	Methylated No. (%)	Unmethylated No. (%)	
	193(59.2)	133(40.8)	
Age[a]			0.852
<50	74(38.3)	47(36.2)	
50-59	75(38.9)	50(38.5)	
>60	44(22.8)	33(25.4)	
Mean ± SD	52.91±9.36	53.27±8.91	0.732
Marriage status[a]			0.649
Single	19(9.9)	11(8.4)	
Married	173(90.1)	120(91.6)	
Educational level[a]			0.257
Primary and below	55(28.6)	40(30.1)	
Junior/senior high school	115(59.9)	70(52.6)	
College and above	22(11.5)	23(17.3)	
Occupation[a]			0.797
Mental worker	46(27.2)	35(29.2)	
Manual worker	57(33.7)	36(30.0)	
Mixed	66(39.1)	49(40.8)	
Family history[a]			0.984
Yes	41(21.7)	27(21.6)	
No	148(78.3)	98(78.4)	
BMI[a]			0.296
<18.5	7(3.6)	4(3.1)	
18.5-22.9	79(40.9)	43(32.8)	
≥23	107(55.4)	84(64.1)	
Mean ± SD	23.90±3.74	24.33±3.94	0.322

[a]Missing values: age 3; marriage status 3; educational level 1; family history 12; occupation 37; BMI 2.

Supplemental Table 3

Univariate and multivariate analyses for the association of environmental factors and breast cancer risk

**Factor**	**Cases No. (%)**	**Controls No. (%)**	**OR_{crude}(95%CI)**	***P*-value**	**OR_{adj}(95%CI)**	***P*-value**
	423	509				
Refined grain (g/day)						
<100	260(74.3)	290(57.4)	1.000		1.000	
≥100	90(25.7)	215(42.6)	0.467(0.347-0.629)	0.000	0.434(0.308-0.610)	**0.000**
Coarse grains(g/week)						
<50	200 (56.8)	247 (48.9)	1.000		1.000	
≥50	152(43.2)	258(51.1)	0.728(0.553-0.957)	0.023	0.755(0.554-1.029)	0.075
Vegetable(g/day)						
<500	283(68.2)	261(52.0)	1.000		1.000	
≥500	132(31.8)	241(48.0)	0.505(0.385-0.662)	0.00	0.657(0.487-0.888)	**0.006**
Fruit(g/week)						
<1000	216(52.4)	291(58.3)	1.000		1.000	
≥1000	196(47.6)	208(41.7)	1.269(0.976-1.651)	0.075	1.569(1.164-2.114)	**0.003**
Fat meat						
No	292(69.2)	341(68.6)	1.000		1.000	
Yes	130(30.8)	156(31.4)	0.973(0.735-1.288)	0.849	0.927(0.677-1.269)	0.637
Beef and mutton (g/week)						
<250	382(90.3)	434(85.6)	1.000		1.000	
≥250	41(9.7)	73(14.4)	0.638(0.425-0.958)	0.030	0.564(0.361-0.883)	**0.012**
Pork(g/week)						
<250	236(57.1)	291(60.1)	1.000		1.000	
≥250	177(42.9)	193(39.9)	1.131(0.866-1.476)	0.366	0.997(0.737-1.349)	0.985
Poultry meat (g/week)						
<250	375(90.8)	426(88.2)	1.000		1.000	
≥250	38(9.2)	57(11.8)	0.757(0.491-1.168)	0.209	0.711(0.443-1.140)	0.156
Freshwater fish(times/month)						
<1	172(41.3)	222(43.7)	1.000		1.000	
≥1	244(58.7)	286(56.3)	1.101(0.847-1.431)	0.472	1.154(0.857-1.555)	0.346
Seafood(times/month)						
<1	378(91.3)	421(83.2)	1.000		1.000	
≥1	36(8.7)	85(16.8)	0.472(0.312-0.713)	0.000	0.528(0.325-0.857)	**0.010**
Soybean(times/week)						
<1	184(44.6)	201(39.7)	1.000		1.000	
≥1	229(55.4)	305(60.3)	0.820(0.630-1.067)	0.140	0.794(0.590-1.069)	0.129
Milk(times/week)						
<3	310(74.0)	300(59.8)	1.000		1.000	
≥3	109(26.0)	202(40.2)	0.522(0.394-0.692)	0.000	0.487(0.349-0.679)	**0.000**
Egg(per week)						
≤3	177(42.0)	229(45.0)	1.000		1.000	
>3	244(58.0)	280(55.0)	1.127(0.869-1.463)	0.367	0.957(0.711-1.288)	0.771

Dessert(times/week)						
<2	315(74.6)	347(68.8)	1.000		1.000	
≥2	107(25.4)	157(31.2)	0.751(0.562-1.003)	0.052	0.744(0.534-1.036)	0.080
Coffee						
No	394(93.4)	470(92.5)	1.000		1.000	
Yes	28(6.6)	38(7.5)	0.879(0.530-1.458)	0.617	0.627(0.345-1.140)	0.126
Tea						
No	354(84.1)	431(85.7)	1.000		1.000	
Yes	67(15.9)	72(14.3)	1.133(0.790-1.626)	0.498	0.96(0.634-1.473)	0.874
Soda						
No	404(96.4)	478(94.1)	1.000		1.000	
Yes	15(3.6)	30(5.9)	0.592(0.314-1.115)	0.105	0.607(0.298-1.235)	0.168
Juice						
No	387(92.6)	467(91.9)	1.000		1.000	
Yes	31(7.4)	41(8.1)	0.912(0.561-1.483)	0.711	0.715(0.417-1.226)	0.223
Fried food (times/month)						
<1	291(69.1)	382(76.2)	1.000		1.000	
≥1	130(30.9)	119(23.8)	1.434(1.071-1.920)	0.015	1.247(0.899-1.729)	0.186
Smoked food(times/month)						
<1	234(85.4)	375(78.8)	1.000		1.000	
≥1	40(14.6)	101(21.2)	0.635(0.425-0.948)	0.026	0.583(0.366-0.929)	**0.023**
Allium food(times/week)						
<4	294(69.7)	231(45.7)	1.000		1.000	
≥4	128(30.3)	275(54.3)	0.366(0.279-0.480)	0.000	0.428(0.317-0.579)	**0.000**
Garlic(times/week)						
<4	50(16.0)	221 (43.7)	1.000		1.000	
≥4	263(84.0)	285 (56.3)	0.175(0.124-0.246)	0.000	0.228(0.157-0.332)	**0.000**
Sauerkraut(times/week)						
<2	263(62.2)	285(56.1)	1.000		1.000	
≥2	160(37.8)	223(43.9)	0.778(0.598-1.012)	0.061	0.821(0.607-1.110)	0.200
Pickles (times/week)						
<2	185(43.8)	235(46.7)	1.000		1.000	
≥2	237(56.2)	268(53.3)	1.123(0.866-1.457)	0.381	1.357(1.010-1.824)	**0.043**
Canning fruit(times/month)						
<1	343(81.9)	438(86.2)	1.000		1.000	
≥1	76(18.1)	70(13.8)	1.386(0.973-1.976)	0.071	1.290(0.967-1.918)	0.209
Canning meat (times/month)						
<1	390(92.6)	472(94.0)	1.000		1.000	
≥1	31(7.4)	30(6.0)	1.251(0.744-2.103)	0.399	1.048(0.581-1.893)	0.875
Overnight food (times/week)						
≤3	239(57.2)	350(70.4)	1.000		1.000	
>3	179(42.8)	147(29.6)	1.783(1.357-2.343)	0.000	1.934(1.422-2.632)	**0.000**
Sports						
No	270(64.4)	270(53.7)	1.000		1.000	
Yes	149(35.6)	233(46.3)	0.639(0.490-0.834)	0.001	0.490(0.362-0.665)	**0.000**
Smoking						

Never	367(87.0)	449(88.4)	1.000		1.000	
Ever	55(13.0)	59(11.6)	1.140(0.770-1.689)	0.511	1.220(0.771-1.932)	0.396
Alcohol						
Never	362(87.4)	439(92.0)	1.000		1.000	
Ever	52(12.6)	38(8.0)	1.659(1.068-2.579)	0.024	1.495(0.910-2.455)	0.112
Mammary gland disease history						
No	261(65.4)	425(84.5)	1.000		1.000	
Yes	138(34.6)	78(15.5)	2.881(2.097-3.958)	0.000	2.412(1.678-3.465)	**0.000**
Gynecologic surgery						
No	263(62.2)	320(63.1)	1.000		1.000	
Yes	160(37.8)	187(36.9)	1.041(0.797-1.359)	0.768	1.164(0.857-1.581)	0.332
Menstrual regularity						
Yes	342(82.2)	458(90.7)	1.000		1.000	
No	74(17.8)	47(9.3)	2.108(1.425-3.119)	0.000	1.891(1.218-2.936)	**0.005**
Pregnant times						
<3	221(53.0)	305(61.5)	1.000		1.000	
≥3	196(47.0)	191(38.5)	1.416(1.088-1.844)	0.010	1.433(1.055-1.945)	**0.021**
Breast massage						
No	185(46.7)	361(74.0)	1.000		1.000	
Yes	211(53.3)	127(26.0)	3.268(2.462-4.337)	0.000	3.746(2.720-5.159)	**0.000**
Estrogenic medication history						
No	394(94.3)	497(97.8)	1.000		1.000	
Yes	24(5.7)	11(2.2)	2.747(1.329-5.676)	0.006	2.180(0.985-4.824)	0.054
Endocrine medication history						
No	406(97.4)	498(98.4)	1.000		1.000	
Yes	11(2.6)	8(1.6)	1.687(0.672-4.233)	0.266	1.204(0.446-3.254)	0.714
IUD(intra uterine device)						
No	105(25.1)	157(30.9)	1.000		1.000	
Yes	313(74.9)	351(69.1)	1.332(0.996-1.782)	0.053	1.443(1.040-2.002)	**0.028**
Oral contraceptive pill						
No	363(87.1)	457(90.1)	1.000		1.000	
Yes	54(12.9)	50(9.9)	1.360(0.904-2.046)	0.141	1.267(0.794-2.022)	0.320
Menopause status						
No	191(46.2)	227(44.6)	1.000		1.000	
Yes	222(53.8)	282(55.4)	0.936(0.721-1.214)	0.617	0.678(0.500-0.919)	**0.012**
Mammary gland hyperplasia						
No	332(79.4)	488(96.3)	1.000		1.000	
Yes	86(20.6)	19(3.7)	6.666(3.978-11.169)	0.000	6.013(3.416-10.582)	**0.000**
Psychological stress score						
≤15	74(17.5)	163(32.5)	1.000		1.000	
16-33	224(53.1)	253(50.4)	1.950(1.404-2.708)	0.000	1.909(1.316-2.770)	**0.001**
＞33	124(29.4)	86(17.1)	3.176(2.153-4.685)	0.000	3.070(1.976-4.769)	**0.000**
Health care products						
No	244(58.5)	358(72.3)	1.000		1.000	
Yes	173(41.5)	137(27.7)	1.853(1.405-2.444)	0.000	1.685(1.224-2.320)	**0.001**
Bra wearing						
rarely	112(26.9)	152(30.6)	1.000		1.000	
general	304(73.1)	345(69.4)	1.196(0.896-1.596)	0.225	1.213(0.853-1.726)	0.282

OR_{crude}, odds ratio generated by univariate logistic regression; OR_{adj}, odds ratio generated by multivariate logistic regression adjusted by education, occupation, marital status, and family history of cancer; 95% CI, 95% confidence interval.

Note:

Soybean includes soybean milk, bean curd, processed soybean products.

Seafood includes shrimp, crab, snail and shellfish.

Sports: We defined "Yes" as ≥1 time/week in the last year. The sports includes yangge dance, hiking, run ning, swimming, rope skipping, fitness activities, gymnastics, yoga, qigong, Taijiquan, football, basketball, volleyball, badminton.

Breast massage: breast massage or kneading to promote milk discharge and secretion in lactation.

Health care products: We defined "Yes" as taking the following health care products ≥3 times/week for m ore

than 2 consecutive months in the last year, including donkey-hide gelatin, honey, royal jelly, propolis, fish oil, spirulina, protein powder, etc.

Menstrual cycle: regular (cycle length: 22–38 days within the previous 3 months) with no use of any regular (daily/weekly) medication; irregularity: get no periods, get periods too frequently, have unpredictable menstrual bleeding, or have painful periods.

Supplemental Table 4

Association of environmental exposures and *DUSP1* methylation in breast tumour DNA

Factor	*DUSP1* methylation status		Univariate analysis	
	**Methylated**	**Unmethylated**	**OR_{crude} (95% CI)**	***P*-value**
Menstrual regularity				**0.012**
No	54(72.0)	21(28.0)	1.000	
Yes	134(55.8)	106(44.2)	0.487(0.277-0.856)	
Menopause status				0.219
No	76(63.9)	43(36.1)	1.000	
Yes	112(56.9)	85(43.1)	0.746(0.467-1.191)	
Breast massage				0.645
Yes	85(57.0)	64(43.0)	1.000	
No	96(59.6)	65(40.4)	1.112(0.708-1.748)	
Oral contraceptive				0.179
No	159(57.6)	117(42.4)	1.000	
Yes	32(68.1)	15(31.9)	1.570(0.813-3.032)	
IUD (intra uterine device)				0.340
No	57(63.3)	33(36.7)	1.000	
Yes	134(57.5)	99(42.5)	0.784(0.475-1.294)	
Mammary gland hyperplasia				0.081
No	157(57.3）	117(42.7)	1.000	
Yes	34(70.8)	14(29.2)	1.810(0.929-3.526)	
Mammary gland disease history				0.196
No	122(57.8)	89(42.2)	1.000	
Yes	63(65.6)	33(34.4)	0.718(0.435-1.186)	
Coarse grains(g/week)				0.316
≤50	103(62.0)	63(38.0)	1.000	
>50	72(56.2)	56(43.8)	0.786(0.492-1.257)	
Vegetable(g/day)				0.614
≤500	122(61.0)	78(39.0)	1.000	
>500	68(58.1)	49(41.9)	0.887(0.558-1.412)	
Soybean(times/week)				**0.007**
≤1	72(51.8)	67(48.2)	1.000	
>1	119(66.9)	59(33.1)	1.877(1.189-2.962)	
Fruit(g/week)				**0.021**
≤1000	89(67.9)	42(32.1)	1.000	
>1000	100(54.9)	82(45.1)	0.576(0.360-0.920)	
Allium food(times/week)				0.253
≤4	132(61.4)	83(38.6)	1.000	
>4	58(54.7)	48(45.3)	0.760(0.474-1.217)	
Fat meat				0.464
Yes	64(62.1)	39(37.9)	1.000	
No	129(57.8)	94(42.2)	0.836(0.518-1.350)	
Beef and mutton(g/week)				0.458
<250	110(58.2)	79(41.8)	1.000	
≥250	78(62.4)	47(37.6)	1.192(0.750-1.894)	
Poultry meat(g/week)				0.313
<250	99(57.2)	74(42.8)	1.000	
≥250	88(62.9)	52(37.1)	1.265(0.801-1.996)	
Sea-fish(times/month)				0.668
<1	108(58.1)	78(41.9)	1.000	
≥1	84(60.4)	55(39.6)	1.103(0.705-1.726)	

Seafood(times/month)				0.786
<1	163(59.7)	110(40.3)	1.000	
≥1	30(57.7)	22(42.3)	0.920(0.505-1.678)	
Egg(per/week)				0.281
≤3	69(55.6)	55(44.4)	1.000	
>3	124(61.7)	77(38.3)	1.284(0.815-2.022)	
Milk				0.982
No	120(59.4)	82(40.6)	1.000	
Yes	53(59.6)	36(40.4)	1.006(0.605-1.672)	
Sauerkraut(times/week)				0.335
<2	81(56.2)	63(43.8)	1.000	
≥2	112(61.5)	70(38.5)	1.244(0.798-1.941)	
Tea				0.221
Yes	26(68.4)	12(31.6)	1.000	
No	167(58.0)	121(42.0)	0.637(0.309-1.313)	
Fried food(times/month)				0.539
<1	124(60.5)	81(39.5)	1.000	
≥1	69(57.0)	52(43.0)	0.867(0.549-1.368)	
Psychological stress score				0.869
≤15	44(61.1)	28(38.9)	1.000	
16-33	101(58.0)	73(42.0)	0.880(0.502-1.544)	0.657
>33	48(60.8)	31(39.2)	0.985(0.512-1.896)	0.965
Canning fruit(times/month)				0.935
<1	125(57.9)	91(42.1)	1.000	
≥1	31(58.5)	22(41.5)	1.026(0.558-1.887)	
Canning meat(times/month)				0.527
<1	178(59.7)	120(40.3)	1.000	
≥1	15(53.6)	13(46.4)	0.778(0.357-1.693)	
Overnight food(times/week)			0.315	
≤3	118(57.0)	89(43.0)	1.000	
>3	74(62.7)	44(37.3)	1.268(0.798-2.017)	
Smoke				0.926
Never	169(59.3)	116(40.7)	1.000	
Ever	24(58.5)	17(41.5)	0.969(0.498-1.884)	
Alcohol				0.736
Never	165(59.4)	113(40.6)	1.000	
Ever	18(56.2)	14(43.8)	0.881(0.421-1.842)	
Sports				0.439
Yes	60(56.6)	46(43.4)	1.000	
No	132(61.1)	84(38.9)	1.205(0.752-1.931)	

OR_{crude}, odds ratio generated by univariate logistic regression; 95% CI, 95% confidence interval.

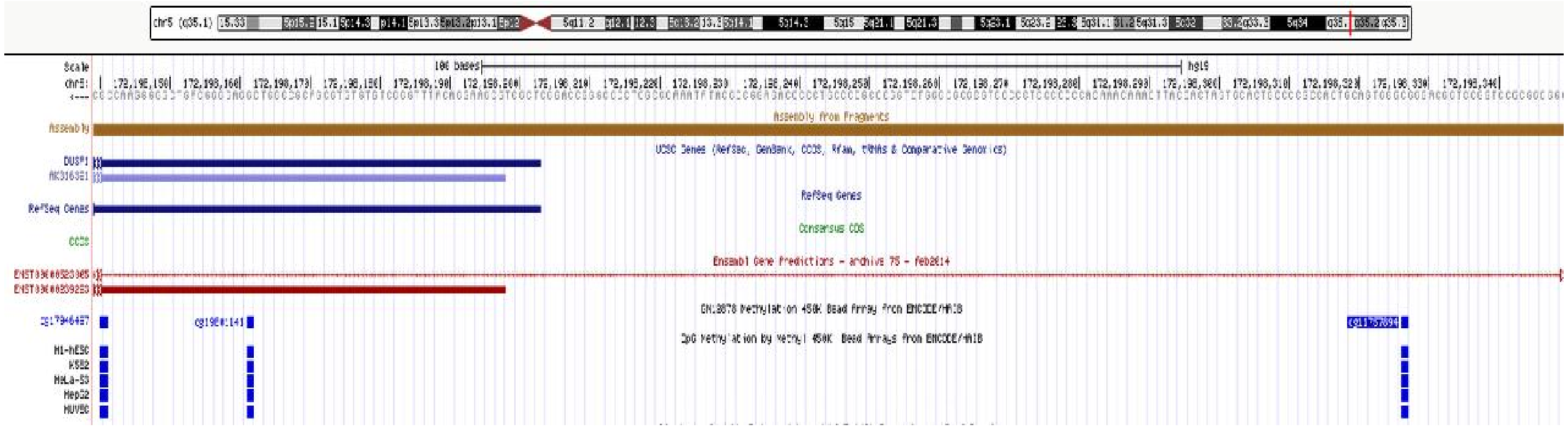

Supplemental Figure 1

Methylation detecting region (range=chr5:172198165-172198336, 171bp) of *DUSP1* gene by Methylation-sensitive high-resolution melting analysis
